# Management of emergent hypertension in Herat Regional Hospital, Afghanistan

**DOI:** 10.1186/1749-8090-8-S1-O253

**Published:** 2013-09-11

**Authors:** MN Faqiryar

**Affiliations:** 1 Herat Regional Hospital, Herat City, Afghanistan

## Background

Hypertensive emergency is an emergent case which all prompt consideration should be taken. Due to control of hypertension less cases of emergent hypertension is seen nowadays; so the data from April 2012 to Dec 2012 in internal ward of the regional hospital shows 305 cases (113 males and 192 females) recorded aged between 16 - 90 years with the average of 49 years. Number of admitting days in hospital was between 2 to 6 days. The mean rate days of hospitalization were 3 days. Although blood pressure is usually strikingly elevated (diastole pressure > 130 mmHg) emergency include hypertensive encephalopathy, hypertensive nephropathy, intra cranial hemorrhage, aortic dissention, preeclampsia, eclampsia, pulmonary edema, unstable angina or MI.

## Methods

The goal of treatment in emergent hypertensive is to reduce BP no more than 25% within an hour and forward the BP to 160/100 mmHg within 2-6 hours to avoid the risk of serious morbidity or death.

The IV medications for hypertensive emergencies are available in our hospital are;

1. Labetalol: 20 mg / 2 minutes bolus then 40 – 80 mg at 10 minutes interval up to 300 mg total. Infusion 2mg / minutes until satisfactory result occurs.

2. Hydralazine: 10- 15 mg at 30 minutes interval.

3. Furosemide: 20 – 40 mg.

## Result

1. Unfortunately injection of Nitroprusside, Enaloprilat, Nicardipine and Nitroglycerin are not available.

2. 190 cased discharged with oral antihypertensive drugs.

3. 16 cases died.

4. 15 cases referred to abroad.

5. 84 cases left hospital without prior permission.

6. Unfortunately half of the cases come to hospitals when CVA or TIA occurs.

## Conclusion

1. Due to insufficient system of data collecting, I have fewer tips about the prognosis of the patients.

2. The only IV antihypertensive drugs we have are Labetalol and Hydralazine.

3. If we were supplied with other IV antihypertensive drugs, we surly can get good results.

4. Unfortunately poor prognosis is seen in elderly hypertensive patients.

